# Supplementary material for: Diagnostic Performance of On-Site Computed Tomography Derived Fractional Flow Reserve on Non-Culprit Coronary Lesions in Patients with Acute Coronary Syndrome
Source: Life (Basel). 2022 Nov 8;12(11):1820. doi: 10.3390/life12111820 (PMC9698642; doi:10.3390/life12111820)
Supplement: Supplementary file 1 [file life-12-01820-s001.zip › Supplementary_Material_revised_ahres_et_al.pdf]

Supplementary Materials

Detailed description of dobutamine stress echocardiography protocol

Rationale behind the use of the 16-segment ventricular model during the procedure: The imperceptible nature of the endocardial excursion and the thickening of the tip of the apex.

Dobutamine infusion protocol: Titrated in 3-minute intervals from 5 to 10, 20, 30, 40 or 50 µg/kg/minute.

Atropine: Intravenous administration up to 2 mg (only if age-predicted submaximal heart frequency was not achieved).

Contrast agent: Intravenous administration, Sonovue (Bracco, Italy). Only in patients with poor image quality.

Definition of ischemia during the procedure: New or worsening wall motion abnormality in ≥2 contiguous myocardium segments, as well as biphasic response.

Endpoints other than ischemia: Maximum dose of pharmacological agents; Achievement of at least age-predicted submaximal (85%) target heart rate; Severe chest pain; Ventricular tachycardia/fibrillation; Patient's request.

Coronary supplying areas

Left anterior descending artery: apical septal; mid. inferoseptal; apical, mid, and basal anterior; apical lateral; apical inferior; mid. and basal anteroseptal.

Left circumflex artery (right dominance): mid. and basal anterolateral.

Left circumflex artery (left dominance): mid. and basal inferolateral; mid. and basal inferior; basal inferoseptal; mid. and basal anterolateral.

Right coronary artery (right dominance): mid. and basal inferolateral; mid. and basal inferior; basal inferoseptum.

Right coronary artery in the case of left dominance: 0 segment in the case of left dominance.

Segmental scores: 1=normokinesia; 2=hypokinesia; 3=akinesia; 4=dyskinesia; 0=not adequately visible segment.

Interpretation of the images: By two independent echocardiographer. In cases of discordant result, final decision has been made by the expert echocardiographer (Peter Andrassy)

Supplementary Table S1 Invasive characteristics of the investigated non-culprit lesions.

	**N=89**
LAD (N, %)	52 (58.4)
LCx (N, %)	15 (16.9)
RCA (N, %)	22 (24.7)
Right dominance (N, %)	61 (89.7)
MLDS% by QCA (%, at the time of the primary PCI)	51.0±8.1
MLDS% by QCA (%, at the time of the staged invasive coronary angiography)	44.9±8.2
FFRi value	0.83±0.08
FFRi determined as positive (N, %)	35 (39.3)
NCLs with LVEF<50% (N,%)	43 (48.3)

Abbreviations: FFRi – invasive fractional flow reserve; LAD – left anterior descending coronary artery; LCx – left circumflex coronary artery; LVEF – left ventricular ejection fraction; MLDS% - degree of minimal lumen diameter stenosis in percentages; NCL – non-culprit lesion; QCA – quantitative coronary angiography; RCA – right coronary artery

Supplementary Table S2 Baseline global and regional echocardiographic findings at rest and at peak stress, respectively.

**Global (patient-based) parameters**	**N=68**
WMA at rest (N, %)	56 (82.4)
Global WMSI at rest	1.27±0.21
Global WMSI at peak stress	1.34±0.28
Global ΔWMSI	0.06±0.27
Contrast enhancement (N, %)	2 (2.9)
Heart rate at rest (1/min)	65.1±8.8
Heart rate at peak stress (1/min)	147.7±10.1
Target heart rate achieved (N, %)	63 (92.6)
**Regional (vessel-based) parameters**	**N=89**
Regional WMSI at rest	1.06±0.18
Regional WMSI at peak stress	1.31±0.50
Regional ΔWMSI	0.25±0.47
DSE determined as positive (N, %)	27 (30.3)

Abbreviations: DSE – dobutamine stress echocardiography; WMA – wall motion abnormality; WMSI – wall motion score index; ΔWMSI – the difference between rest and peak wall motion score index (rest minus peak)

Supplementary Table S3 2 by 2 contingency table of CT-FFR measurement compared with FFRi (Panel A) and DSE (Panel B).

Overall, CT-FFR showed a moderate match with FFRi (χ^2=17.527, p<0.001; kappa=0.427, p<0.001); however, it did not have any association with DSE (χ2=1.996, p=0.158; kappa=0.149, p=0.158).

Panel A

	**FFRi +**	**FFRi -**	
**CT-FFR +**	18	6	24
**CT-FFR -**	17	48	65
	35	54	N=89

CT-FFR versus FFRi: accuracy: 74% (95% CI: 64%-83%); sensitivity: 51% (95% CI: 34%-69%); specificity: 89% (95% CI: 77%-96%); PPV: 75% (95% CI: 57%-87%); NPV: 74% (95% CI: 66%-80%); PLR: 4.6 (95% CI: 2.0-10.5); NLR: 0.55 (95% CI: 0.38-0.78)

Panel B

	**DSE +**	**DSE -**	
**CT-FFR +**	10	14	24

**CT-FFR -**	17	48	65
	27	62	N=89

CT-FFR versus DSE: accuracy: 65% (95% CI: 54%-75%); sensitivity: 37% (95% CI: 19%-58%); specificity: 77% (95% CI: 65%-87%); PPV: 42% (95% CI: 27%-58%); NPV: 74% (95% CI: 67%-80%); PLR: 1.64 (95% CI: 0.84-3.22); NLR: 0.81 (95% CI: 0.59-1.12)

Abbreviations: CI – confidence interval; CT-FFR – computed tomography-based fractional flow reserve; DSE – dobutamine stress echocardiography; FFRi – invasive fractional flow reserve; NLR – negative likelihood ratio; NPV – negative predictive value; PLR – positive likelihood ratio; PPV – positive predictive value

Supplementary Table S4 2 by 2 contingency table of CT-FFR measurement relative to FFRi in the subgroups according to ACS type (STEMI: Panel A; NSTE-ACS: Panel B).

Panel A

	**FFRi +**	**FFRi -**	
**CT-FFR +**	12	4	16
**CT-FFR -**	8	33	41
	20	37	N=57

CT-FFR in STEMI: accuracy: 79% (95% CI: 66%-89%); sensitivity: 60% (95% CI: 36%-81%); specificity: 89% (95% CI: 75%-97%); PPV: 75% (95% CI: 53%-89%); NPV: 80% (95% CI: 70%-88%); PLR: 5.55 (95% CI: 2.06-14.97); NLR: 0.45 (95% CI: 0.26-0.78)

Panel B

	**DSE +**	**DSE -**	
**CT-FFR +**	6	2	8
**CT-FFR -**	9	15	24

	15	17	N=32

CT-FFR in NSTE-ACS: accuracy: 66% (95% CI: 47%-81%); sensitivity: 40% (95% CI: 16%-68%); specificity: 88% (95% CI: 64%-99%); PPV: 75% (95% CI: 42%-93%); NPV: 63% (95% CI: 52%-72%); PLR: 3.40 (95% CI: 0.80-14.38); NLR: 0.68 (95% CI: 0.43-1.06)

Abbreviations: CI – confidence interval; CT-FFR – computed tomography-based fractional flow reserve; FFRi – invasive fractional flow reserve; NLR – negative likelihood ratio; NPV – negative predictive value; NSTE-ACS – Non-ST-elevation acute coronary syndrome; PLR – positive likelihood ratio; PPV – positive predictive value; STEMI – ST-elevation myocardial infarction**Supplementary Table S5** Correlation analysis of CCTA-based anatomical and functional parameters (row) with FFRi and regional WMSI at peak stress during DSE (column).

	Lesion length		MLDS%		CT-FFR value		CT-FFR drop	
	r	P	r	p	r	p	r	p
FFRi value	-0.09	0.402	-0.26	0.015	0.54	<0.001	-0.54	<0.001
Regional WMSI at peak stress	-0.08	0.478	-0.16	0.139	-0.05	0.622	0.04	0.715

Abbreviations: CCTA – coronary computed tomography angiography; CT-FFR – computed tomography-based fractional flow reserve; CT-FFR drop – CT-FFR value difference between proximal and distal measuring point (proximal minus distal); FFRi – invasive fractional flow reserve; MLDS% - degree of minimal lumen diameter stenosis in percentages; WMSI – wall motion score index

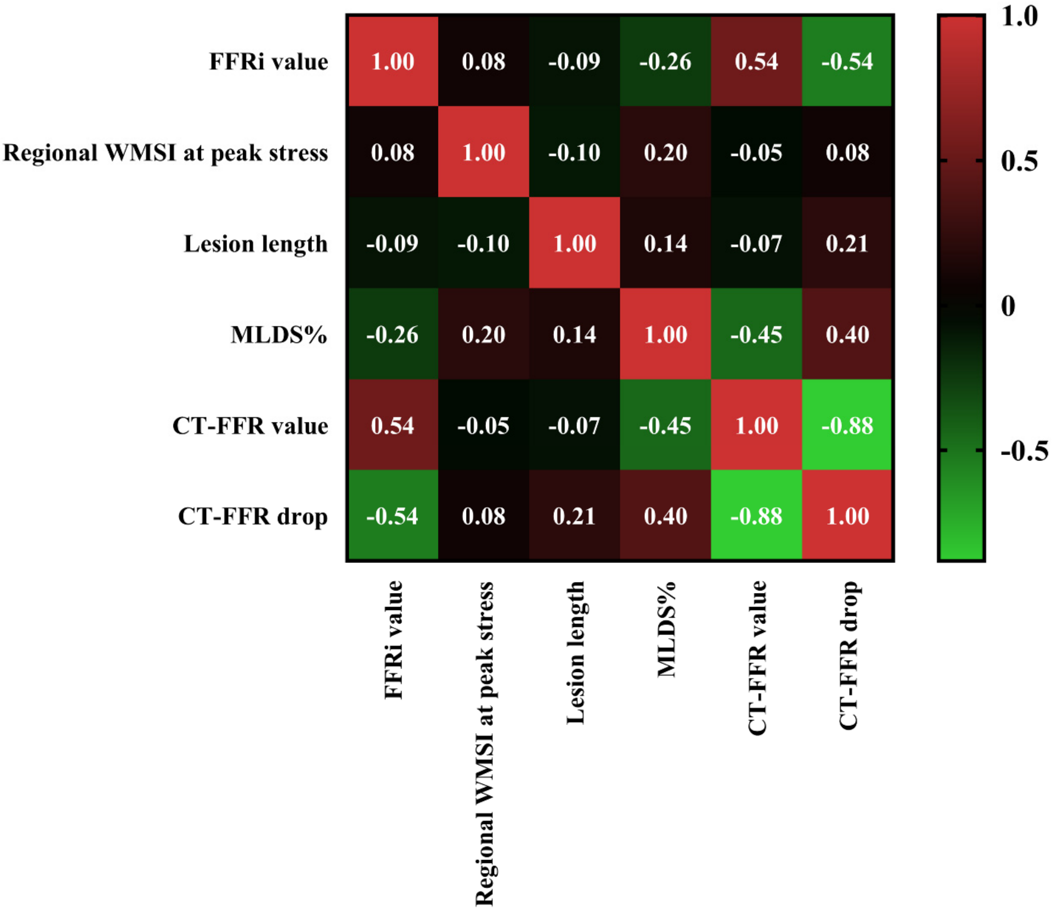

Supplementary Figure S1 Correlation analysis between multimodal functional (CCTA, FFRi and DSE) parameters: a heatmap.

Abbreviations: CCTA – coronary computed tomography angiography; CT-FFR – computed tomography-based fractional flow reserve; CT-FFR drop – CT-FFR value difference between proximal and distal measuring point (proximal minus distal); DSE – dobutamine stress echocardiography; FFRi – invasive fractional flow reserve; MLDS% - degree of minimal lumen diameter stenosis in percentages; WMSI – wall motion score index
