# Supplementary figures and images for: Diagnostic Performance of On-Site Computed Tomography Derived Fractional Flow Reserve on Non-Culprit Coronary Lesions in Patients with Acute Coronary Syndrome
Source: Life (Basel). 2022 Nov 8;12(11):1820. doi: 10.3390/life12111820 (PMC9698642; doi:10.3390/life12111820)

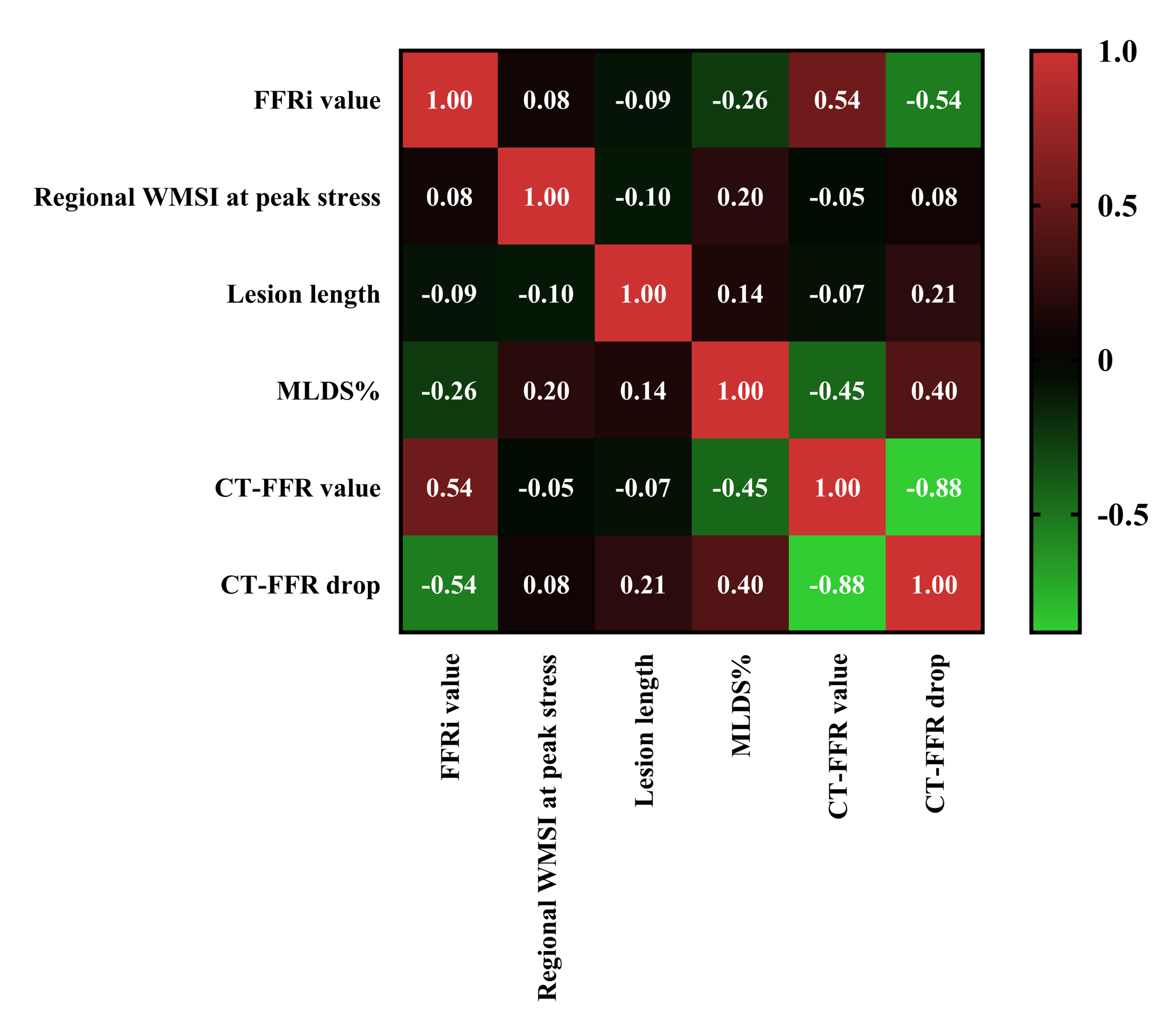

Supplement: Supplementary file 1 [file life-12-01820-s001.zip › Supplementary_Figure_S1.tif]
